# Football Brutalities

**Published:** 1889-05-25

**Authors:** 


### Editor's Letter-Box.

[Our Correspondents are reminded that prolixity is a great bar to publication, and that brevity of style and conciseness of statement greatly facilitate early insertion.]

### FOOTBALL BRUTALITIES.

I have read with considerable interest your remarks on "Football Brutalities" in a recent number of the *Hospital*. You would seem, however, to have ignored the fact that almost all the accidents which take place among football players are the result of an imperfect idea of the game, nor does any accident you refer to appear to have taken place in a school match. How is it, sir, I should like to ask, that we never hear of such accidents among such clubs as Preston or Blackheath? I do not think it is very difficult to solve the question. It is because there is no need for casualties; neither do they happen among clubs which play football according to the constituted rules. If any remedy be wanting, let it be that everyone shall be prohibited playing unless he be a certificated player, and let heavy fines be inflicted on those clubs in which accidents take place. No one more deeply deplores these casualties than I, but I think it both absurd and unfair to condemn on this account the rules or the play of the game, especially when those who might be expected to show us a good example are the very ones who are the greatest enthusiasts themselves—I mean some of our hospital teams.—Yours, etc., A Young Schoolmaster.

